# Network toxicology and molecular docking analyses on strychnine indicate CHRM1 is a potential neurotoxic target

**DOI:** 10.1186/s12906-022-03753-4

**Published:** 2022-10-17

**Authors:** Jialin Dai, Jiangjin Liu, Maoxin Zhang, Yanni Yu, Jie Wang

**Affiliations:** 1 School of Forensic Medicine, Guizhou Medical University, Guiyang, Guizhou Province China; 2 Department of technology, Zhongshan branch of Liupanshui Public Security Bureau, Liupanshui, Guizhou Province China

**Keywords:** Network toxicology, Molecular docking, Strychnine, CHRM1

## Abstract

**Background:**

Improper use of strychnine can cause death. The aim of this study was to identify and evaluate toxic mechanisms of action associated with active compounds in strychnine using a network toxicology approach, and explore potential pathogenic targets.

**Methods:**

In the present study, strychnine target and central nervous system-related gene set were established using the Traditional Chinese Medicine Systems Pharmacology (TCMSP) database and four disease gene databases (Genecards, OMIM, PharmGkb, TTD). An “ingredient-target” interactive active network map was constructed using Cytoscape software (version 3.8.0). Functional enrichment analysis was performed based on the hub genes. A protein-protein interaction network was constructed using STRING database. The pharmacokinetics (ADMET) properties of strychnine were evaluated using SwissADME tool. Molecular docking was performed using Autodock Vina to explore the interactions between the active compounds and the target protein.

**Results:**

Five strychnine toxicity-related components and a gene set of 40 genes were obtained. GO and KEGG analyses showed that Strychnine acts on the central nervous system through G protein-coupled receptor signaling pathway. Analysis of “ADMET” related parameters showed a high gastrointestinal tract absorption of (S)-stylopine and isobrucine and the compounds could cross the blood brain barrier. CHRM1 was selected as a key gene in strychnine toxicity. Molecular docking results showed that the co-crystalized ligands did not form hydrogen bond with CHRM1. (S)-stylopine had the highest binding affinity (binding energy = − 8.5 kcal/mol) compared with the other two compounds.

**Conclusion:**

Network toxicology and molecular docking reveal the toxicity mechanisms of strychnine active compounds. The findings showed that CHRM1 is a potential neurotoxic target. (S)-stylopine showed stronger neurotoxic effect compared with the other ligands.

## Introduction

*‘Maqianzi’* herb is used in Chinese folk medicine. It is used as a herbal remedy for rheumatism, musculoskeletal injuries and limb paralysis after processing to reduce its toxicity [1]. In addition, *maqianzi* exhibits excellent anti-tumor effect on various tumors [2, 3]. Although herbal medicine is used worldwide for its benefits in treatment of diseases, side effects and toxicity limit the widespread use of herbal medicine [4]. A previous study reported cases of people who had died after taking herbal medicine (this was later confirmed to be caused by strychnine) to promote bone healing [5]. The victims presented with significant tremor and muscle spasms prior to death. Autopsy showed that the limbs had spastic flexion, the feet were significantly pronated and extended, and cyanosis was observed on the nail beds. The histopathological manifestations were not specific, and the main diagnosis was strychnine poisoning.

**Fig. 1:**
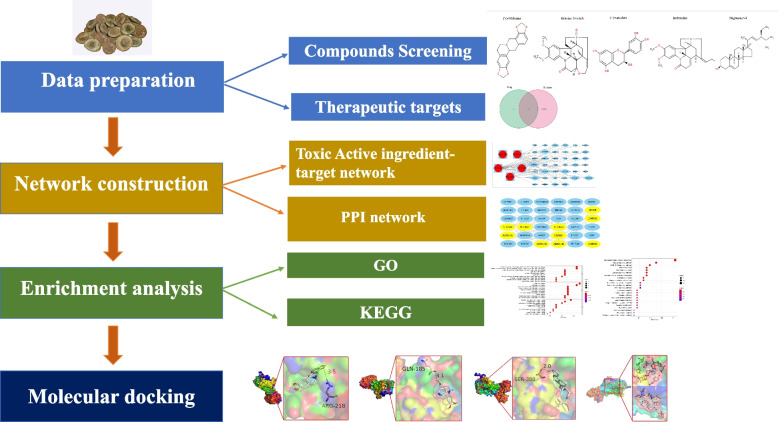
The flowchart of technical strategy in current study

Studies report that alkaloids are the major bioactive components of maqianzi plant. The plant contains 1.0–1.4% of strychnine and brucine which are responsible for the toxicity effects [6]. These alkaloids can cause severe central nervous system toxicity by increasing neuronal activity and excitability, leading to increased muscular activity. The cause of death is mainly respiratory arrest secondary to respiratory muscle spasms [7]. However, as a Chinese herbal medicine, it is challenging to evaluate its toxicity mechanism from a single point of view because of its multi-component, multi-target, and multi-pathway characteristics. Therefore, a network toxicology approach was used in the current study to explore the network relationships between strychnine toxic components and their corresponding targets using several online database resources. Mechanism of strychnine toxicity was explored from multiple perspectives, and the mechanism of action of strychnine in the central nervous system was evaluated using molecular docking analysis. CHRM1 was identified as the key gene responsible for toxicity of strychnine, thus it is a potential neurotoxic target. The experimental design of the present study is shown in Fig. 1.

## Materials and methods

### Retrieval of strychnine toxic compounds and neurological-related genes

Data on the main ingredients of strychnine were retrieved from the TCMSP database (https://tcmspw.com/) [8] including active compounds and their target genes. Various compounds used in traditional Chinese medicine and their corresponding pharmacokinetic indicators were obtained using the keyword “Maqianzi”. The criteria for compound screening were oral bioavailability (OB) above 30% and drug-like (DL) index > 0.18. Strychnine toxic compounds were obtained by uploading the compounds from TCMSP database directly into the Comparative Toxicogenomics Database (http://ctdbase.org/,CTD) for toxicity analysis. A search of the related target genes was conducted for each compound in TCMSP. Pharmacokinetics “ADMET” properties of strychnine were evaluated using SwissADME tool (http://www.swissadme.ch/) [9].

Neurological injury-related genes were retrieved from four databases namely: Genecards database (https://www.genecards.org/) [10], OMIM database (https://omim.org/) [11], PharmGkb database (https://www.pharmgkb.org/) [12], and TTD database (http://db.idrblab.net/ttd/) [13]. Further, a neurological-related gene set was established by selecting intersecting genes in a Venn diagram generated using the search results from the four databases.

Strychnine toxic component target and neural-associated gene sets were obtained by selecting the intersection between the target genes set for toxic components of strychnine set and the neural-associated gene set.

### Disease target network and analysis of function functional enrichment analysis

The intersecting components obtained between the active components of strychnine and neurological-related targets were imported into Cytoscape3.8.0 [14], for construction of a regulatory network of the toxic components in Strychnine. DAVID webserver (https://david.ncifcrf.gov) was used to perform gene ontology (GO) and Kyoto Encyclopedia of Genes and Genomes (KEGG) pathway analysis [15]. The “ggplot2” package in R software version 4.0.2 was used to generate figures.

### Construction of protein-protein interaction (PPI) network and identification of core targets

The Strychnine toxic component target and neural-associated gene sets were uploaded into the STRING database (https://string-db.org/) (version 11.0), to explore the interactive relationships among these genes. The results were imported into the Cytoscape for visualization of the network. The MCODE plugin was used to perform sub-network analysis using the following parameters: “Degree=2, Node score cutoff=0.2, k-core=2” to identify core targets in the network.

### Structure for the molecule ligands and the crystal structures of the core protein

Key genes obtained from the sub-network were used for molecular docking analysis. The 3D structures for the molecule ligands ((S)-stylopine, isobrucine and stigmasterol) were retrieved from the PubChem database (https://pubchem.ncbi.nlm.nih.gov/). Further, the receptor protein encoded by the target gene was obtained from the UniProt database (https://www.uniprot.org/) and the 3-D structures and co-crystallized ligand, CHRM1 (PDB-ID: 6WJC) were retrieved from the Protein Data Bank (PDB) database (https://www.rcsb.org/).

### Molecular docking

The protein structures were optimized using Pymol software (vision: 2.4.0) by removing water molecules and other small molecule ligands before conducting molecular docking. Autodock Tools (vision:1.5.6) software was hired to remove water molecules, remove ligands and added hydrogen atoms to receptor target. The target protein was then used as a receptor, and the active pharmaceutical ingredients were used as ligand molecules. The active binding site of the molecular docking were identified based on the coordinates of the co-crystallized ligand. The Grid-box coordinates and size were set according to the active pocket of the target protein. Autodock Vina was used for docking of the receptor protein with the small molecule ligands. Analysis and visualization were performed using PyMol software.

## Result

### Screening of toxic active compounds and potential targets

**Fig. 2:**
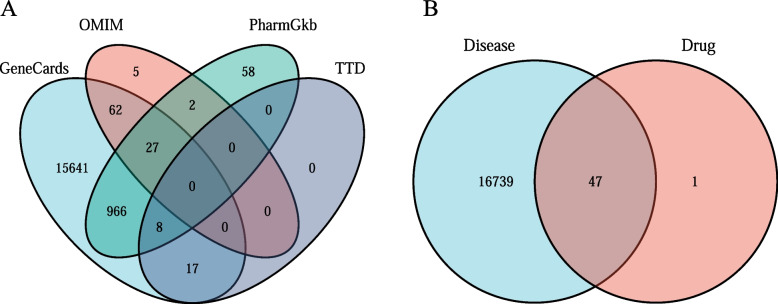
Screening of toxic active compounds and potential targets. **A** Neurological-related gene from four database, respectively, with duplicates removed and combined with the search results, obtained disease gene set. **B** Intersection of the corresponding target genes in the five toxic components with disease-related genes to obtain drug-disease gene sets of 40 genes

A total of 13 strychnine compounds were identified from TCMSSP database based on the criteria OB ≥ 30%, DL ≥ 30.18. Toxicity information for five strychnine compounds including (S)-stylopine, isobrucine, brucine N-oxide, stigmasterol and (+)-catechin was obtained from the CTD database. A total of 65 drug target genes were selected from the CTD database. Further, neurological-related genes were retrieved from Genecards, OMIM, PharmGkb, and TTD databases. Duplicates were removed and the results were combined with the above results, and a total of 16,786 neurological-related genes were obtained (Fig. 2A). The final set of strychnine target genes and neurological-related genes was obtained by selecting the intersection of compound target genes and disease-associated genes (Fig. 2B).

### Active ingredient-target network construction for evaluation of strychnine toxicity

**Fig. 3:**
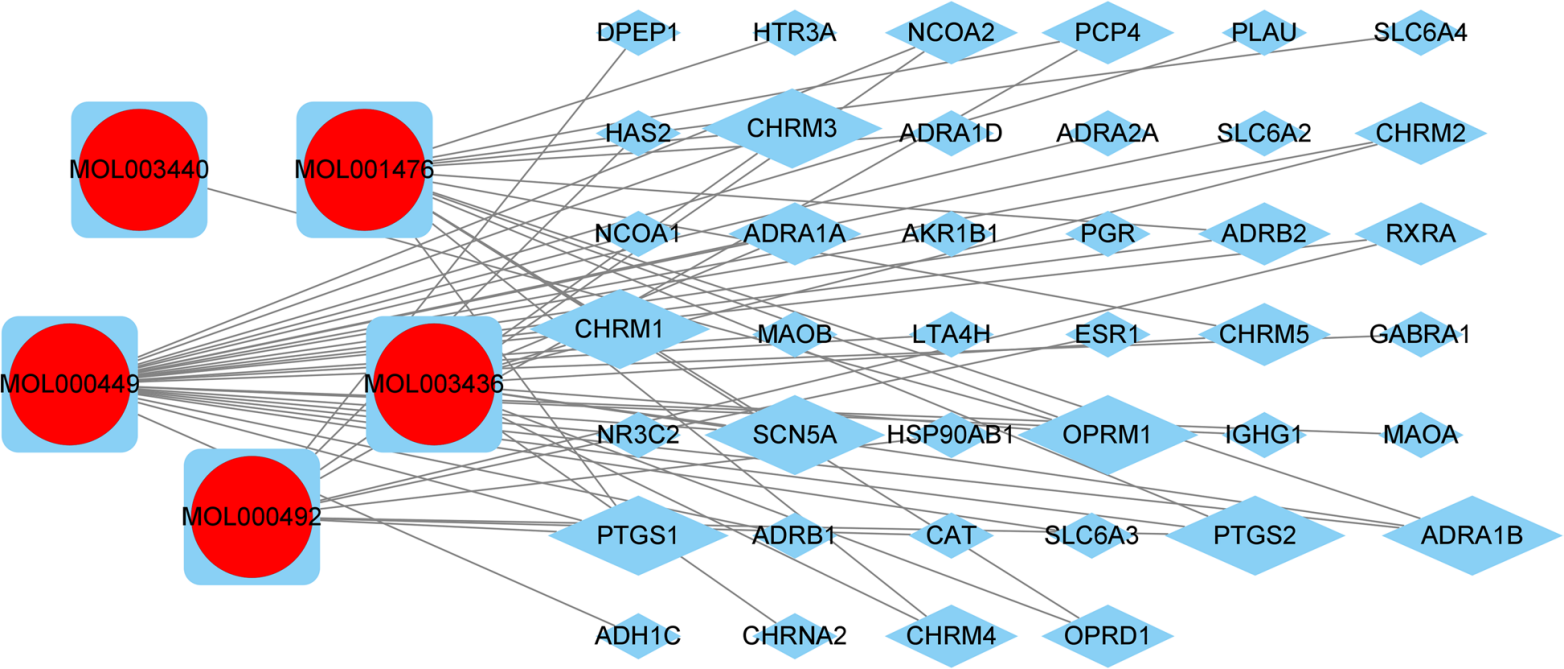
Toxic Active ingredient-target network. Of these, the red circles represent strychnine toxic active ingredients, and the blue diamonds are target genes

The five toxic active ingredients (Table 1) and target genes of strychnine were imported into Cytoscape for network visualization to explore the relationship between the toxic active ingredients and the corresponding target genes (Fig. 3). Calculation of the linkage value (Degree value) gives the number of target genes directly linked to the component. A high linkage value indicate that the gene has a key position in the network. The ranking results showed that PTGS1, CHRM3, CHRM1, SCN5A, PTGS2, ADRA1B, and OPRM1 were the most important genes in the network.

**Table 1:**
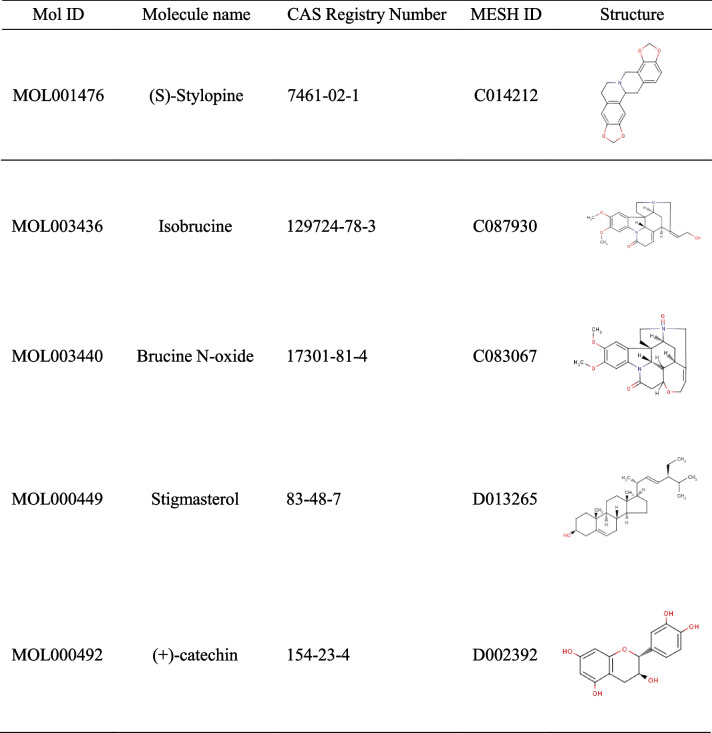
Toxic candidates in strychnine

### “ADMET” properties of strychnine active compounds

The “ADMET” properties of the active ingredients were evaluated to determine the mechanism of strychnine toxicity. The results showed that the gastrointestinal tract absorption (GI absorption) of (S)-stylopine and isobrucine was higher compared with stigmasterol, co-crystallized ligand 1 and Co-crystallized ligand 2. In addition, (S)-stylopine was able to cross the blood brain barrier permeant (BBB permeant). In addition, the findings showed that (S)-stylopine was an inhibitor of cytochrome P450 enzyme (isozymes: CYP1A2, CYP3C4, CYP2C19, and CYP2D6) (Table 2).

**Table 2:** ADMET information of (S)-Stylopine, Isobrucine and Stigmasterol

Compounds	(S)-Stylopine	Isobrucine	Stigmasterol	Co-crystal lig1	Co-crystal lig2
PubChem CID	440,583	3,081,763	5,280,794	NA	NA
GI absorption	High	High	Low	Low	Low
BBB permeant	Yes	Yes	No	No	No
CYP2D6 inhibitor	Yes	Yes	No	No	No
CYP3A4 inhibitor	Yes	No	No	Yes	No
CYP1A2 inhibitor	Yes	No	No	No	No
CYP2C19 inhibitor	Yes	No	No	No	No

### PPI network and identified core genes

**Fig. 4:**
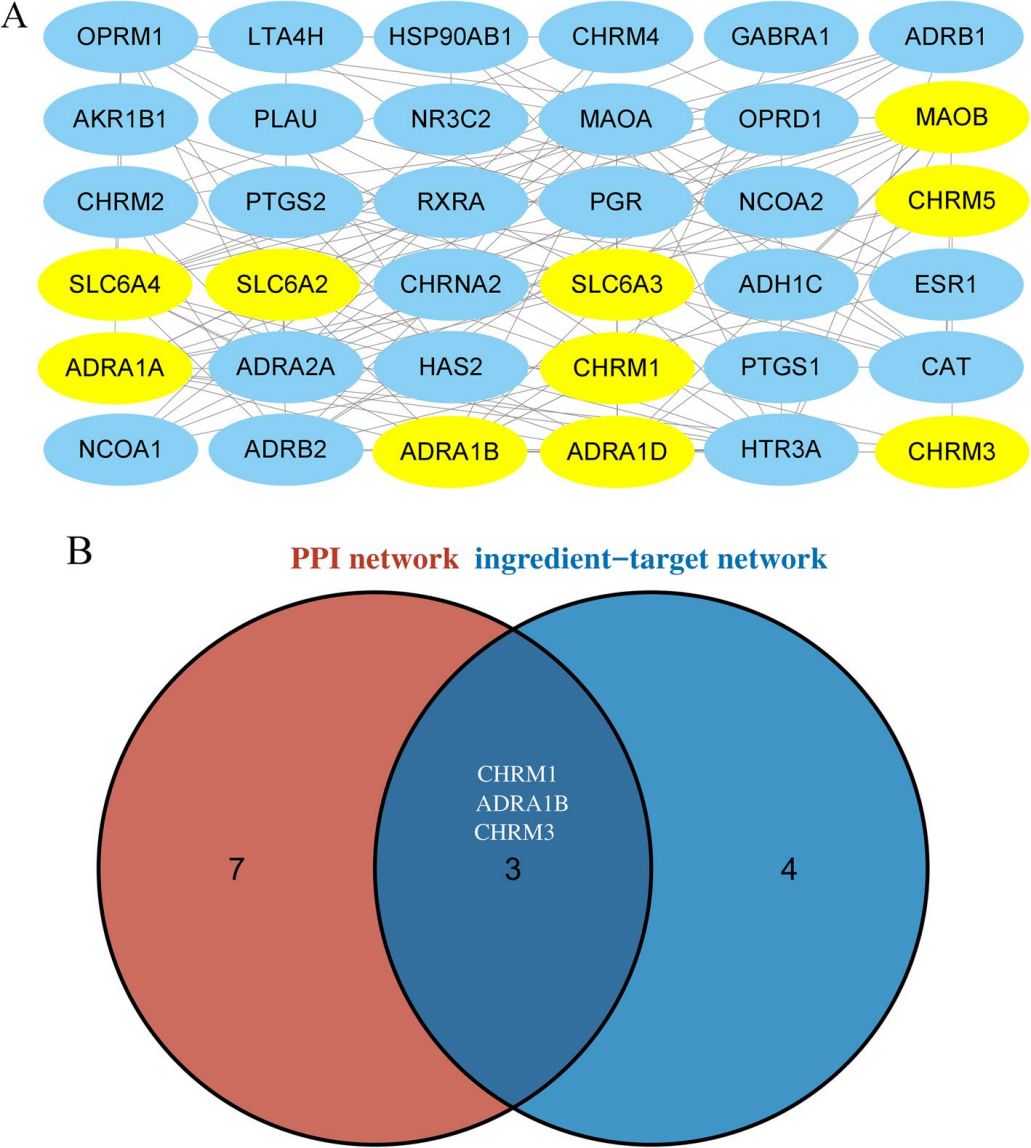
The protein-protein interaction (PPI) network, module analysis, and hub gene selection. **A** Results of subnet module analysis of the PPI network via MCODE plug-in, the yellow oval represents hot genes. **B** Screening of the key genes by taking an intersection of the two key subnetworks

The target genes were imported into Cytoscape and the MCODE plug-in was used with the parameters were set to: degree = 2, node score cutoff = 0.2, k-core = 2, to explore the interactions of the proteins encoded by the target genes. A total of 10 hub genes were obtained (Fig. 4A). The key genes from the toxic active ingredient-target network were combined with the hub genes from the PPI network and the results showed that CHRM1 was a target gene for strychnine active compounds (Fig. 4B).

### Functional enrichment analysis of strychnine target genes

**Fig. 5:**
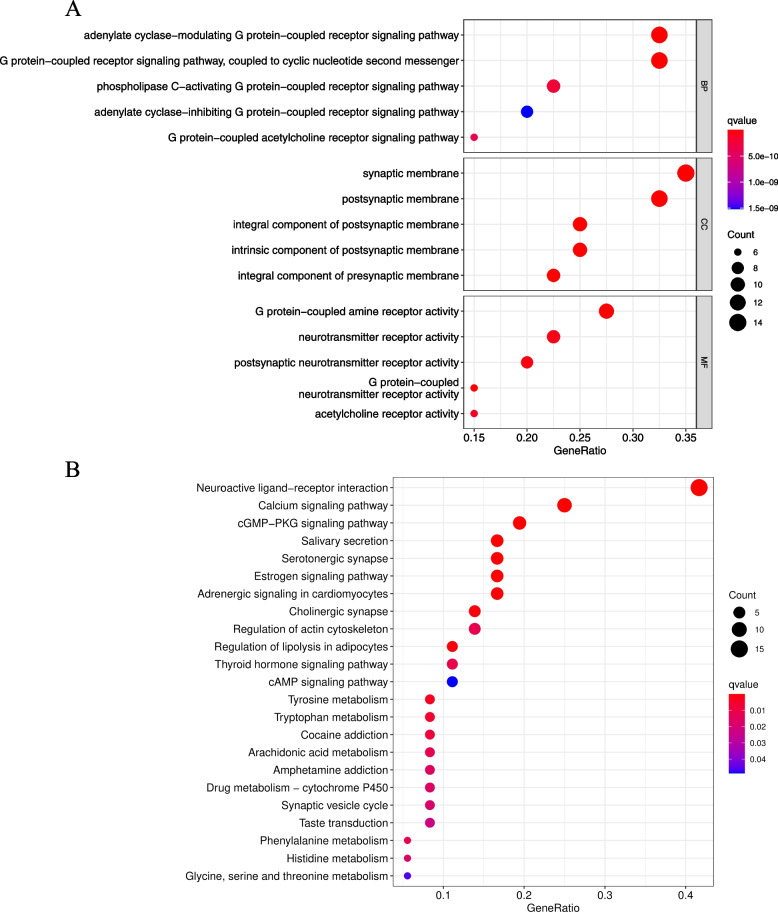
Function enrichment analysis. **A** Detailed information relating to changes in the biological processes (BP), cellular components (CC), and molecular functions (MF) of target genes through the GO enrichment analyses. **B** The KEGG pathway analysis of target genes

The biological processes (BP), cell components (CC), molecular function (MF) and KGGG of 40 genes were evaluated to gain further insight on the biological functions of the identified target genes related to the toxic active ingredient of strychnine. GO results showed a total of 576 significantly enriched terms. The top 10 terms, using *P* < 0.05 as the threshold, are presented in Fig. 5A. The GO results indicated that these target genes played key roles in G protein-coupled receptor signaling pathway and synaptic membrane. KEGG enrichment analysis was performed and 23 significantly enriched terms, including neuroactive ligand-receptor interaction, calcium signaling pathway and cGMP-PKG signaling pathway were identified. The significant terms are presented in Fig. 5B. The results from GO and KEGG analyses indicated that Strychnine compounds act on central nervous system through multiple pathways. CHRM1 was significantly enriched in these important pathways, implying that the target gene plays an important role in neurological symptoms as a result of strychnine poisoning.

### Molecular docking of toxic active compounds to CHRM1-encoded protein

**Fig. 6:**
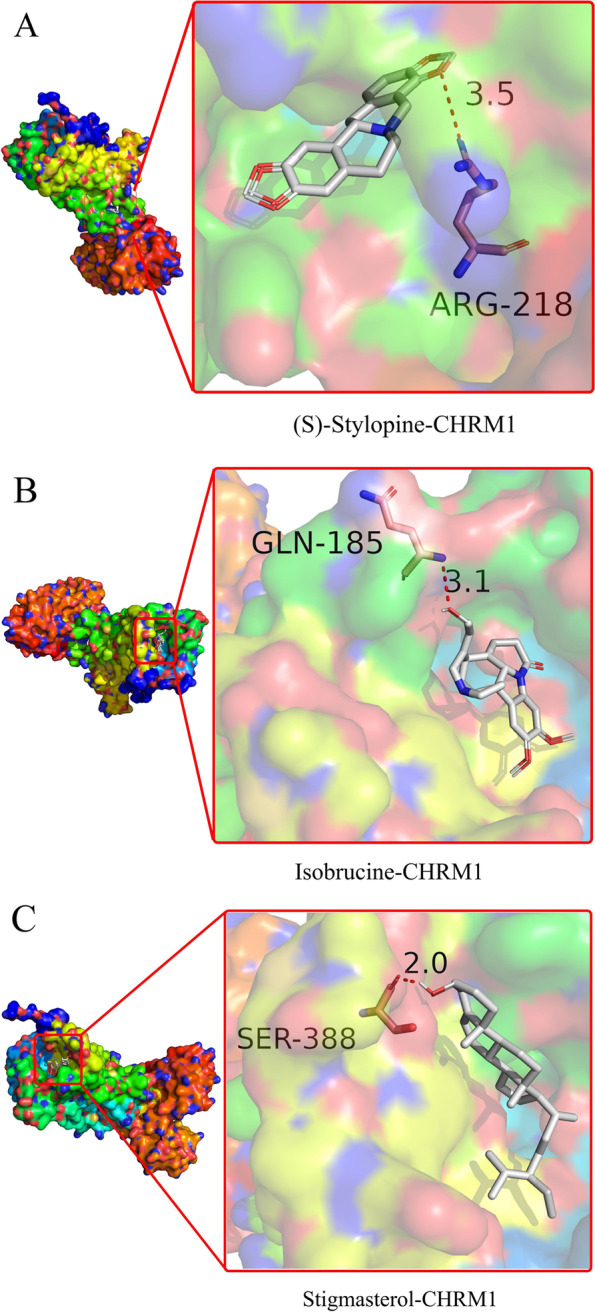
Molecular docking of key target compounds, the bottom diagram shows the 3D structure of the ligand and the receptor, while the top diagram shows the 3D structure of the receptor surface and the ligand. **A** Docking process of (S)-Stylopine with CHRM1. **B** Docking process of Isobrucine with CHRM1. **C** Docking process of Stigmasterol with CHRM1. * The red dotted line indicates the H-bond force between the compound and CHRM1protein

The most important gene, CHRM1, was selected for molecular docking. Three active compounds targeting the CHRM1 protein were obtained by TCMSP dataset analysis. The three compounds were (S)-stylopine, isobrucine and stigmasterol. Results from molecular docking analysis showed that all three active compounds were binding at the active pocket of the CHRM1 protein. Further, the active ingredient of strychnine showed good binding affinity to the key CNS target protein CHRM1. (S)-stylopine formed an interaction with Arg218 of CHRM1 on the A chain through a hydrogen bond. Isobrucine compound showed an interaction with Gln185 of the A chain of CHRM1 through a hydrogen bond. Stigmasterol formed a hydrogen bond with Ser388 in the A chain of CHRM1 protein. The 2D interactions picture was incomplete, so a 3D image was generated (Fig. 6A-C). The binding energies for the three compounds are presented in Table 3.

**Table 3:** Molecular docking of toxic active compounds to CHRM1-encoded proteins

Compounds	Targets	Affinity (kcal/mol)	PDB ID	H-bond	Box size (x,y,z)
(S)-Stylopine	CHRM1	−8.5	6WJC	1	(40,40,40)
Isobrucine	CHRM1	−7.8	6WJC	1	(40,40,40)
Stigmasterol	CHRM1	−8.2	6WJC	1	(40,40,40)
Co-crystal ligand 1	CHRM1	−7.5	6WJC	0	(40,40,40)
Co-crystal ligand 2	CHRM1	−7.1	6WJC	0	(40,40,40)

**Fig. 7:**
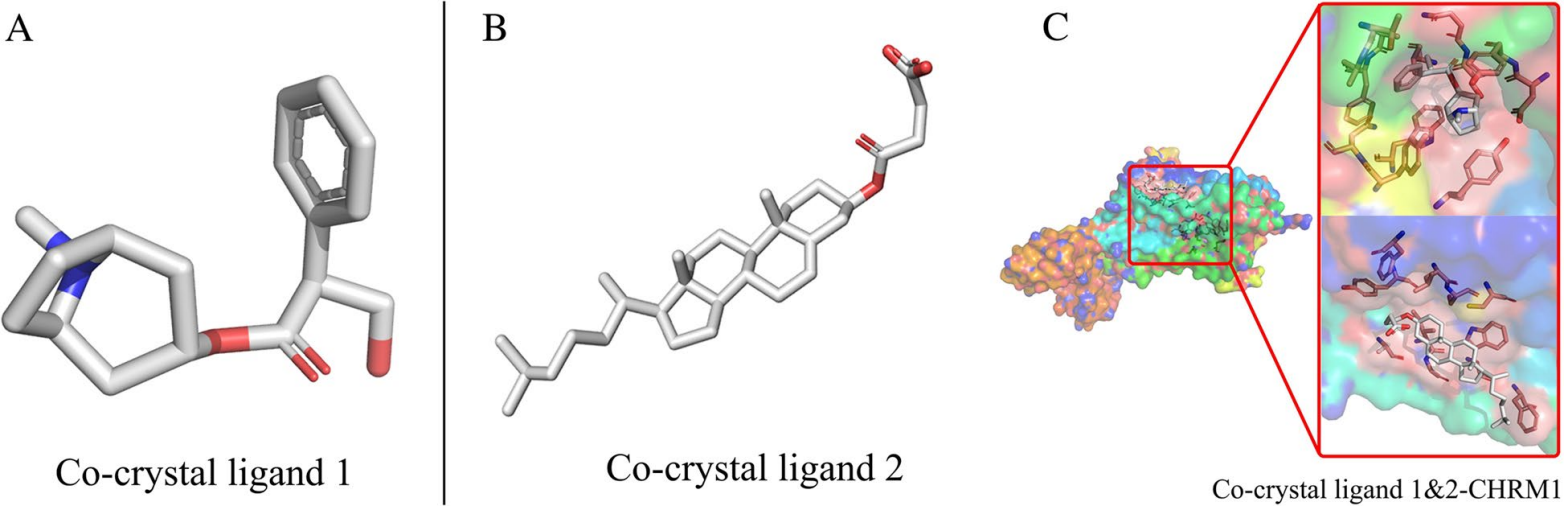
Molecular docking of two eutectic ligands of CHRM1 protein. **A** Co-crystal ligand1 of CHRM1. **B** Co-crystal ligand2 of CHRM1. (C) Docking process of two eutectic ligands with CHRM1

Molecular docking results of the co-crystalized ligands showed no hydrogen bonds (Fig. 7A-C) and the binding energy were lower compared with that of strychnine compounds (Table 3).

## Discussion

Maqianzi herb is used in Chinese folk medicine. It is commonly used for anaesthesia and treatment of traumatic injuries, pains, paralysis and tumors [1, 16–18]. Maqianzi contains strychnine and brucine that have high toxicity [6]. Strychnine poisoning is not common. Strychnine mainly exerts its effects as a competitive antagonist of the inhibitory neurotransmitter glycine at receptors in the spinal cord, brain stem and higher centers. It blocks postsynaptic receptors of the inhibitory neurotransmitter glycine and presynaptic action inhibiting release of the inhibitory neurotransmitter in the spinal cord and motoneurons. Strychnine toxicity causes tonic convulsions and death directly through spinal paralysis or respiratory or cardiac arrest [5]. A history of ingestion of herbal remedies and visible neurological symptoms should be explored for strychnine poisoning, which is evaluated to define the cause and manner of death under supervision of forensic medical practitioners. Although the pharmacological effects of the main compounds in maqianzi have been fully explored, the specific toxicity mechanism of these compounds have not been fully elucidated.

In the present study, 13 active compounds extracted from maqianzi were retrieved from TCMSP database. Toxicity information for the 5 strychnine components was obtained from the CTD database. A strychnine target neurological-related gene set that comprised 40 target genes was obtained by analyzing the active components from the 5 ingredients of strychnine. A toxic active ingredient-target network for strychnine toxicity was constructed, and 7 genes were identified as the most important genes in the network. The results showed that different components were linked to different targets, indicating a multi-component, multi-target action of strychnine.

Key genes from the toxic active ingredient-target network and the hub genes from the PPI were further analyzed and the findings showed that CHRM1 was the target gene for strychnine toxicity.

The results from functional enrichment analysis showed that the identified target genes in the toxic active ingredient of strychnine were mainly implicated in neuroactive ligand-receptor interaction, and calcium signaling pathway. This implies that the compounds directly increase neuronal activity and excitability, leading to increased muscular activity. These findings are consistent with findings from previous studies. The top 5 most significant GO (BP) terms indicated that the identified target genes are involved in G protein-coupled receptor signaling pathway and phospholipase C-activating G protein-coupled receptor signaling pathway. Cholinergic muscarinic receptors are G protein-coupled receptors (GPCRs) that modulate several vital functions of the central and peripheral nervous systems. These receptors are classified into M1–M5 receptors [19, 20], and they play a role in regulating heart rate, smooth muscle contraction, glandular secretion and several key functions of the central nervous system (CNS) [21]. CHRM1 was involved in the GO terms and KEGG pathways in the present study. Therefore, CHRM1 gene was selected for subsequent analyses and molecular docking between the small molecule and the protein encoded by *chrm1* gene was performed.

Three molecules namely; (S)-stylopine, isobrucine and stigmasterol, were selected as potential ligands for CHRM1 protein. This finding indicates that CHRM1 is a potential neurotoxic target. ADME prediction using SwissADME web server showed that the three molecules exhibited wide distribution and poor metabolic excretion.. Several studies report that strychnine is an antagonist of glycine thus it is effective as a pesticide. The active compounds bind noncovalently to the same receptor (acetylcholine binding protein), preventing the inhibitory effects of glycine on the postsynaptic neuron. The motor neurons are more easily activated, when the inhibitory signals are prevented, resulting in spastic muscle contractions, and ultimately death by asphyxiation [22]. These results are consistent with findings from autopsy of a dead body caused by strychnine poisoning, which exhibited symptoms such as spastic flexion, pronated and extended feet, and cyanosis of nail beds.

Comparison between predicted ligands and natural co-crystalized ligands is an effective means to study and predict the toxic effect of ligands. Therefore, the two co-crystalized ligands were redocked to CHRM1 protein, and the docking results were compared with the results of the three predicted ligands. The findings showed that (S)-stylopine had the highest binding affinity with a binding energy of − 8.5 kcal/mol, followed by stigmasterol and isobrucine. These results indicate that the active ingredients from maqianzi have a good binding affinity to key target protein molecules. The three active compounds formed hydrogen bonds with the target protein whereas co-crystalized ligands did not show hydrogen bond interactions with CHRM1. The molecular docking results were consistent with the network pharmacology screening results, and reliability of network pharmacology was verified by molecular docking.

Further, the ADMET related parameters of the three compounds (such as gastrointestinal absorption rate, whether it can penetrate the blood-brain barrier, etc.) were compared with those of the two co-crystalized ligands of CHRM1. The findings showed that (S)-stylopine had stronger neurotoxic effect compared with the other ligands.

Previous studies report that the CHRM1 is the most important muscarinic receptor in the CNS and is highly expressed in neurons. CHRM1 mediates various cellular responses. It is implicated in cellular responses such as adenylate cyclase inhibition, phosphoinositide degeneration, potassium channel mediation, and modulates several effects of acetylcholine in the central and peripheral nervous system. In addition, CHRM1 is involved in modulation of vagally-induced bronchoconstriction and in secretion of acid in gastrointestinal tract. CHRM1 is a member of neuromuscular presynaptic modulating receptors [23]. Lee et.al reported that CHRM1 is a functional muscarinic receptor that induces intracellular Ca^2+^ signaling in response to Ach in striatal cells [24]. Phospholipase C-activating G protein-coupled receptor signaling pathway is a major signaling transduction pathway modulated by muscarinic receptor activation. The pathway is induced by activation of phospholipase C (PLC) and results in release of inositol trisphosphate (IP3) and diacylglycerol (DAG). Subsequently, IP3 increases intracellular calcium levels from Ca^2+^ stores such as the endoplasmic reticulum (ER) by binding the IP3 receptor and CHRM1 regulates Ca^2+^ release through ER [25]. Giessel et al. reported that M1 muscarinic receptors boost synaptic potentials and calcium influx in dendritic spines by inhibiting postsynaptic SK channels [26]. These findings indicate that the toxic component of strychnine may be mediated through the effect of the CHRM1 gene on G protein-coupled receptor pathway. The compounds ultimately affect synaptic signaling and calcium ion concentrations, leading to a series of abnormal actions, directly causing tonic convulsions and death through spinal paralysis, respiratory or cardiac arrest. The findings of the present showed that CHRM1 is a potential neurotoxic target [22]. However, the exact mechanism of toxicity should be explored through molecular studies.

## Conclusion

In the present study, the toxic active components and molecular mechanisms of strychnine-induced neurological symptoms were explored using network toxicology approaches. The results of the study provide a basis for subsequent studies on strychnine toxicity and provide information on the toxicity mechanism of herbal medicines in forensic toxicology. However, the findings of the present should be validated through experimental studies.

## Data Availability

The datasets supporting the conclusions of this article are available in public databases from TCMSP, CTD, OMIM, GeneCards, CTD, TTD, and PharmGKB.
